# Cost Effective Chemical Oxidative Synthesis of Soluble and Electroactive Polyaniline Salt and Its Application as Anticorrosive Agent for Steel

**DOI:** 10.3390/ma12091527

**Published:** 2019-05-10

**Authors:** Anwar-ul-Haq Ali Shah, Muhammad Kamran, Salma Bilal, Rizwan Ullah

**Affiliations:** 1 Institute of Chemical Sciences, University of Peshawar, Peshawar 25120, Pakistan; anwarulhaqalishah@uop.edu.pk (A.-u.-H.A.S.); kamranics@uop.edu.pk (M.K.); 2 NationalCentre of Excellence in Physical Chemistry, University of Peshawar, Peshawar 25120, Pakistan; srizvi2007@hotmail.com; 3 TU Braunschweig Institute of Energy and Process Systems Engineering, Franz-Liszt-Straße 35, 38106 Braunschweig, Germany

**Keywords:** polyaniline, inverse emulsion polymerization, diesel, corrosion protection, stainless steel

## Abstract

The cost effective synthesis of electroactive polyaniline (PANI) while retaining its desirable properties is one of the most debatable and challenging tasks for researchers in the field. Herein, we report a cost effective inverse emulsion polymerization pathway for the synthesis of soluble and processable PANI salt by using diesel as a novel dispersion medium. Different reaction parameters and their effects on the properties and yield of polyaniline were optimized. The polymer exhibited a highly porous morphology and was found to be stable up to 417 °C. The PANI salt showed good solubility in common solvents, such as chloroform, *N*-Methyl-*2*-pyrrolidone (NMP), dimethyl sulphoxide (DMSO) and in a 1:3 mixtures by volume of 2-propanol and toluene. The coating of the synthesized PANI salt on stainless steel has shown good corrosion resistant behavior in marine water by reducing the corrosion rate to 67.9% as compared to uncoated stainless steel.

## 1. Introduction

Polyaniline (PANI) has been under intense investigation for the last few decades [1], in its different redox states, including pernigraniline, lecuemeraldine, emeraldine base and emeraldine salt. It has been synthesized by different methods including electrochemical [2], chemical oxidative [3] including emulsion [4], and inverse emulsion polymerization techniques [5]. Among these, inverse emulsion polymerization is reported to be more effective because PANI obtained by this method possesses enhanced conductivity [6], solubility in common organic solvents [7], thermal stability, and good processability [8]. 

The properties of the final product considerably rely on different reaction parameters such as the type of oxidant, dopant, non-aqueous phase and vice versa. Particular attention has been paid to the type of organic phase by employing different types of organic dispersion media. Table 1 shows the effects of employing different dispersion media on the properties of PANI. PANI-bearing composites, having good electrical conductivity and good mechanical properties, were synthesized through inverse emulsion polymerization using a toluene and iso-octane mixture as the dispersion medium [9]. Rao, P.S. et al. used chloroform as the dispersion medium and benzoyl peroxide as the oxidant and obtained a thermally stable and soluble PANI [6].

A mixture of toluene and 2-propanol, was used by Shreepathi, S. and Holze, R. for polymerization of aniline, soluble in chloroform, 2:1 mixture of toluene and 2-propanol, NMP and dichloromethane [7,10]. Later on, pure toluene was used as a dispersion medium but the solubility was reduced [11]. Bang et al. synthesized highly bolometric Near Infra Red (NIR) sensitive PANI composites with carbon nanotubes in hydrochloric acid media [12]. Similarly, Sun et al. [13], have used perchloric acid as a medium for the fabrication of PANI nanofibers coated with platinum for applications in fuel cells. The synthesis of PANI/DBSA salt using a DBSA-CTAB mixture as surfactant and toluene as a dispersion media was reported by Calheiros et al. [14]. The synthesized materials were reported to have good electrical conductivity and effectiveness at electromagnetic interference shielding. 

Several other methods using different media have been employed to obtain processable and soluble PANI for various applications but the main problem of production costs associated with these methods, particularly in terms of dispersion mediums, persists. For example the cost of the commonly used but not easily available dispersion mediums such as chloroform (13 USD/L), 2-propanol (25 USD/L), 2-butanol (22 USD/L), toluene (22 USD/L), n-hexane (12 USD/L) and iso-octane (158 USD/L) makes the production of PANI a difficult task from commercial point of view. In recent years, we have reported sophisticated methodologies for the synthesis of PANI salts with desirable properties [4,15]. Herein, as further improvement, we report a cost effective and facile synthetic route for the synthesis of PANI salt with improved solubility, good electrochemical activity and excellent corrosion protection ability by using diesel (0.83 USD/L) as a cost effective and novel dispersion media. It can be observed from Table 1 that the use of diesel can not only reduce the cost of production, but also that the synthesized polyaniline exhibits superior properties.

## 2. Material and Methods

### 2.1. Materials

Analytical grade aniline (Acros organic, Morris, NJ, USA) was double distilled under vacuum and stored under a cold environment. Commercial diesel obtained from Pakistan State Oil (PSO), as a complex mixture of hydrocarbons with carbon numbers in the range C9 and higher, having boiling range 160 to 366 °C, specific gravity of 0.828 and viscosity 3.11 cst at 20 °C, was used as received. Other chemicals like benzoyl peroxide (BPO) (Merck, Kenilworth, NJ, USA), dodecylebenzensulphonic acid (DBSA) (Acros organic, Morris, NJ, USA) and acetone (Sigma Aldrich, St. Louis, MO, USA) were also used as received. Ultra pure (Millipore, Burlington, MA, USA) and natural water of Indian Ocean having an average of 3.5% salinity were used, respectively, for solution preparation and corrosion studies.

### 2.2. Synthesis of Polyaniline Salt

The Polyaniline salt was synthesized following an inverse emulsion polymerization protocol [18]. In a typical experiment 30 mL of diesel, employed as a novel dispersion medium, was taken in a round bottom flask and 2.9 mmol of benzoyl peroxide was added to it followed by addition of 1.5 mmol DBSA and 2.2 mmol of aniline. A white milky emulsion was formed by addition of 30 mL of distilled water to this reaction mixture. The reaction mixture was kept on stirring for 24 h at room temperature. Afterwards, the mixture was put into a separating funnel and 30 mL acetone was added to it. Organic and inorganic phases were separated. Organic phase containing PANI was washed with distilled water several times. After washing with water, 30 mL acetone was added to it in order to break emulsion and green colored PANI was precipitated at the bottom. Then PANI was washed with acetone several times and the obtained product was dried in oven at 60 °C for 4 h. 

### 2.3. Optimization

A number of reactions were carried in the same manner but varying different reaction parameters in order to check the effect of monomer, DBSA, oxidant and solvent on the properties and percentage yield of PANI. The product obtained was further processed for characterization and applications. Different samples synthesized with varying amounts of monomer, dopant, oxidant, and solvents were coded as mentioned in Table 2, Table 3, Table 4 and Table 5. Percentage yield in terms of aniline concentration was calculated by using the following formula [4].

(1) $$\%\;\mathrm{yield}\;\mathrm{of}\;\mathrm{PANI}=\frac{\mathrm{Weight}\;\mathrm{of}\;\mathrm{PANI}}{\mathrm{Weight}\;\mathrm{of}\;\mathrm{aniline}}\times 100$$

### 2.4. Characterization

Since it is important to know about the properties of a material before subjecting it to some applications, different tools were utilized to characterize the synthesized PANI. 

UV/Visible spectra of samples were recorded in chloroform in the range of 300–900 nm by using a Perkin Elmer (Buckinghamshire, UK) spectrophotometer. 

Fourier-transform infrared spectroscopy (FTIR) analysis of powder PANI salt was carried out in the range of 400–4000 cm^−1^ by using a IRAffinity-1S Shimadzu Fourier Transform Infrared Spectrophotometer (Shimadzu, Tokyo, Japan). XRD (X-ray diffractometer) patterns of PANI were achieved by using Cu Kα radiations (λ = 1.5405 Å) JEOL JDX-3532 (JEOL, Tokyo, Japan). SEM micrographs were taken by using a JSM-6490 (JEOL, Tokyo, Japan) electron microscope. Thermal stability of PANI samples were determined by using Perkin Elmer, Diamond series (Waltham, MA, USA) at heat rate of 10 °C/min under nitrogen atmosphere. Electrochemical properties were studied by ALS/DY 2323 Biopotentiostate (ALS, Tokyo, Japan). Cyclic voltammograms were recorded in a glass cell containing three electrode assembly and 0.5 M H_2_SO_4_. PANI salt was dip-coated onto a gold sheet working electrode. Optimum care was made to make sure that every time same amount of polymer is loaded on the electrode. For this purpose, 5.8% PANI salt solutions were always prepared in mixture of chloroform and 2-propanol and gold sheet electrode was dipped into it for one minute. Saturated calomel electrode (SCE) and another gold sheet served as reference electrodes and counter electrode, respectively. Corrosion studies were performed by using Reference 3000 Zero Resistance Ammeter (ZRA) potentiostat/galvanostat (Gamry, Warminster, PA, USA).

### 2.5. Application

Selected PANI salts samples were tested for their corrosion protection ability for stainless steel electrode against SCE in water samples from Indian Ocean. Prior to this experiment, the disc of stainless steel was thoroughly polished with an abrasive paper and washed with acetone, followed by ethanol and water in order to remove any soluble impurities. Due to the physical shape of the steel disc electrode, polymer loading was done through drop coating. PANI solution was prepared in the manner mentioned above for gold sheet electrode and a clear glass rod was dipped in the solution and used to put a drop on disc electrode. After putting only one drop the solvent was allowed to evaporate leaving PANI salt in the form of a smooth thin layer on electrode. 

After drying, the working electrode was transferred to the three electrode cell containing water sample from the Indian Ocean. Tafel plot was plotted using ZRA potentiostat/galvanostat Reference 3000 Gamry (USA). DC105 DC corrosion software was used for this study. Potentiodynamic current density measurements were performed at 25 °C by scanning the potential from −400 mV to +400 mV at a scan rate of 3 mV/s. Corrosion current (*i*_corr),_ corrosion potential (E_corr_), β_A_, β_C_ and corrosion rate (CR, mm/year) was determined by extrapolation of Tafel plot. The experiments were carried out under the same conditions in triplicate for each sample. In order to see the effect of film thickness on corrosion protection performance, PANI films with different number of drops coated on the electrode were studied.

## 3. Results and Discussion

### 3.1. Effect of Different Parameters on % Yield

The % yield of PANI salt was found to be greatly affected by changing different parameters like amount of monomer, oxidant, dopant and solvents. Additional details are given below.

#### 3.1.1. Effect of Amount of Monomer

**Figure 1:**
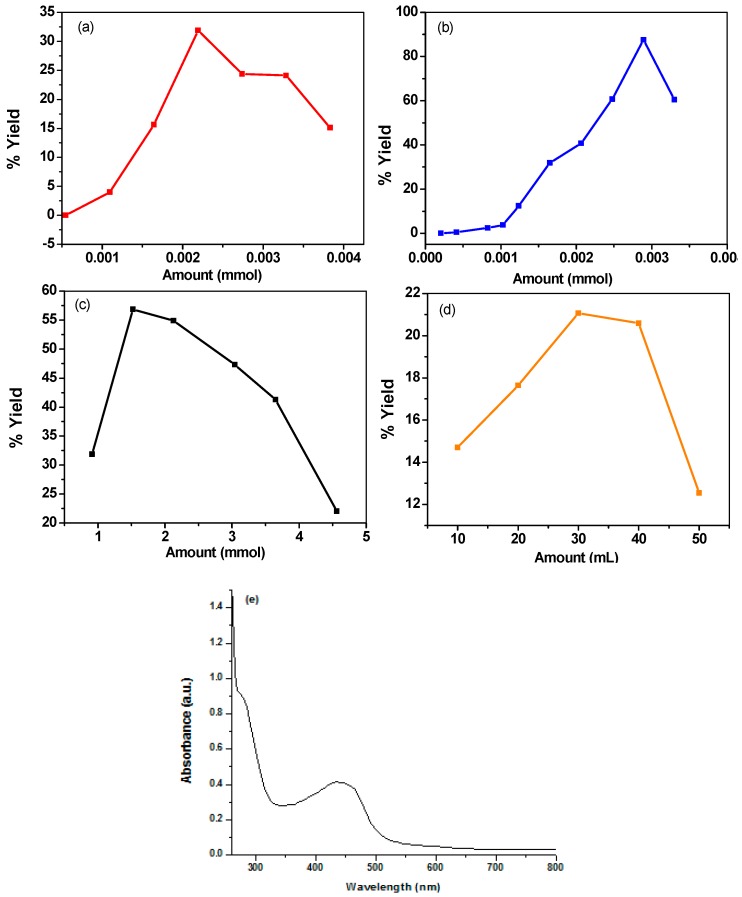
Effect of different reaction parameters on % yield of PANI (**a**) Amount of monomer, (**b**) Amount of Oxidant, (**c**) Amount of dopant in mmol, (**d**) Amount of solvent in mL, and (**e**) UV/Visible spectrum filtrate from product washing indicating the presence of oligomers.

Figure 1a shows the % yield of PANI in terms of amount of aniline in the feed. At a very small amount of aniline, no formation of the PANI occurred and instead some oligomers were formed. The maximum yield was obtained at 2.20 mmol of aniline in the reaction mixture, beyond this a decrease in the % yield of reaction was observed, which can be attributed to the high monomer to oxidant ratio that caused a decrease in the efficiency of the oxidant [19] (Table 2). The sample with maximum yield was labeled as PANI A4.

#### 3.1.2. Effect of Amount of Oxidant

Figure 1b shows the effect of amount of oxidant on the % yield of PANI. At a very low amount of oxidant (0.21 mmol) no polymerization of aniline occurs, presuming that this amount was not sufficient to initiate the oxidation of aniline monomer. Increasing the amount of oxidant causes a gradual increase in the % yield of PANI and maximum yield was obtained at 2.90 mmol of oxidant, after which there was a decrease in the % yield as shown in Table 3. The % yield was found to decrease after a further increase in the amount of oxidant, which could be attributed to the production of excess of radical cations which may result in shorter polymer chain called oligomers. These oligomers were soluble in acetone and removed from the PANI product during the washing [20]. The sample having maximum yield was labeled as PANI B9.

#### 3.1.3. Effect of Amount of Dopant

The effect of amount of dopant on the % yield of PANI is shown in Figure 1c. An increase of % yield was observed when the amount of DBSA was changed from 0.91 mmol to 1.52 mmol which could be due to the insertion of more DBSA into polymer chain, hence increasing its weight. The sample with maximum yield was named as PANI D2. After 1.52 mmol there was a decrease in the yield which may be due to the formation of more micelle having limited number of monomer particles, which results in limited growth of the polymer chain. As a result, small chain polymer formation occurs. This product was soluble in acetone and removed during washing [21]. The filtrate was further analyzed using UV/Visible spectroscopy. The UV/visible spectrum of filtrate is given in Figure 1e. The appearance of peaks in 445 and 279 nm regions supported the formation of oligomers [22,23].

#### 3.1.4. Effect of Amounts of Solvents

Figure 1d shows the effect of amounts of solvents (diesel and water) on the % yield of PANI. The quantity of both the solvents was varied from 10 mL to 50 mL and maximum yield was obtained for 1:1 ratio (30 mL diesel and 30 mL water). The sample obtained at 1:1 ratio of solvents was labeled as PANI S3. Increasing the amount of water as inorganic phase did not favor the inverse emulsion polymerization [10], whereas a further increase in the amount of diesel could reduce the chances of collision between monomer and oxidant particles to produce PANI due to the denser nature of diesel. 

After optimizing all the reaction conditions necessary for the synthesis, an experiment was conducted in which all optimized conditions were applied and the polymer obtained was labeled as PANI salt.

### 3.2. Solubility of the Polymer

**Figure 2:**
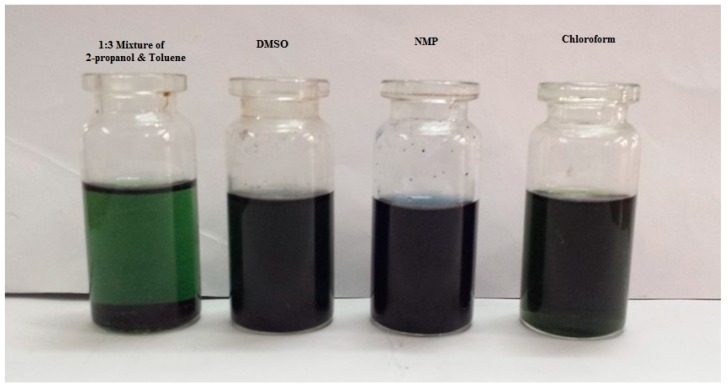
Solutions of PANI salt in different solvents as indicated.

The solubility of PANI salt was checked in different common solvents and was found to be soluble in chloroform, NMP, DMSO and in a 1:3 mixture by volume of 2-propanol and toluene. Better solubility (5.8%) was observed in chloroform. The solubility of PANI in NMP and DMSO can be attributed to the polar nature of these solvents while solubility in chloroform might be due to the fact that the attachments of three electronegative chlorine atoms to carbon makes hydrogen more electropositive which in turn form a hydrogen bond with PANI [4]. The PANI salt was insoluble in pure ethanol, 2-propanol, and toluene but was soluble in 1:3 mixture of 2-propanol and toluene, which may be due to presence of polar and non polar end in 2-propanol. Perhaps the non polar end of 2-propanol interacts with the non polar toluene resulting in a big non polar group which in turn interacts with long non polar alkyl group of DBSA while the polar end of 2-propanol form hydrogen bonds with PANI [24]. The appearance of green color in chloroform and 1:3 mixture of 2-propanol and toluene confirms the doping of DBSA into PANI. The blue color of other PANI solutions suggests its transformation to the base form. This behavior could be due to the solvatochromic effect of PANI and its interaction with solvents characterized by different polarity. Solvatochromism is the property of a chemical substance to change color with the change of solvent polarity. The solvatochromic shift of a chromophore reflects a strong relation between solvent polarity with the absorption and emission spectra [25,26]. The solutions of PANI in different solvents are shown in Figure 2.

### 3.3. UV/Visible Spectroscopy

**Figure 3:**
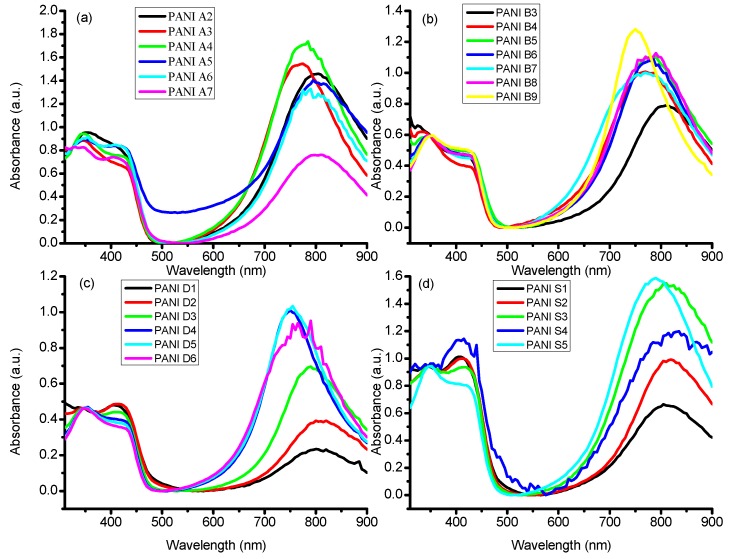
UV/Visible Spectra of different PANI samples indicated in Table 1, Table 2, Table 3 and Table 4. (**a**) Monomer optimized samples (**b**) Oxidant optimized samples (**c**) DBSA optimized samples (**d**) Solvents optimized samples.

The doping effect of DBSA on PANI was investigated through UV/Visible spectroscopic analysis, where chloroform is used as a solvent. The UV/Visible spectra of PANI salts, having different concentrations of monomer, oxidant, dopant and solvent are given in Figure 3a–d, respectively. All the samples give rise to three characteristics absorption bands, with slight variation in the band position, in the range of 341–349, 411–417, and 761–800 nm, which correspond, respectively, to π–π* transition of the benzenoid ring, polaron-π* transition of the quinoid ring and π-polaron transition [16,27,28].

The differences in the peak positions and intensities in the UV/Visible spectra of these samples might be a consequence of the extent of doping and different conjugation length of polymer chains [11,15,29].

**Figure 4:**
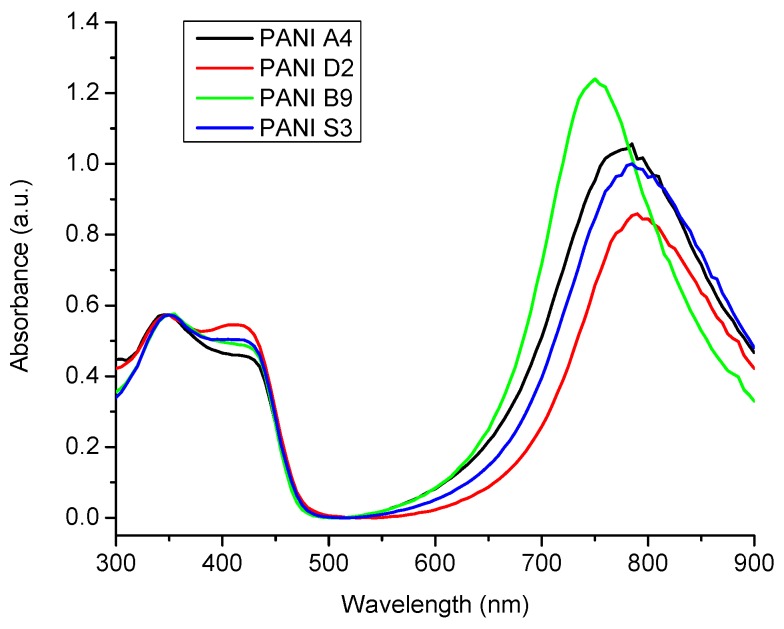
UV/Visible spectra of PANI A4, PANI D2, PANI B9 and PANI S3.

For ready comparison, the UV/Visible spectra of selected samples synthesized with varying amounts of monomer, oxidant, DBSA and solvent are shown in Figure 4. From these spectra it is indicated that PANI has been successfully prepared in the salt form. The presence of two peaks in the visible region for all samples indicates that the samples are collected in the doped form [8]. PANI A4, PANI B9, PANI D2, and PANI S3 showed almost same UV/Visible spectra, however, a blue shift can be seen in the red region peak of PANI B9. This might be due to over oxidation of aniline due to presence of more oxidant units which resulted in shorter polymer chain. Due to smaller chain formation, steric hindrance between dopant and polymer chain increases and hence transition may become difficult [30].

**Figure 5:**
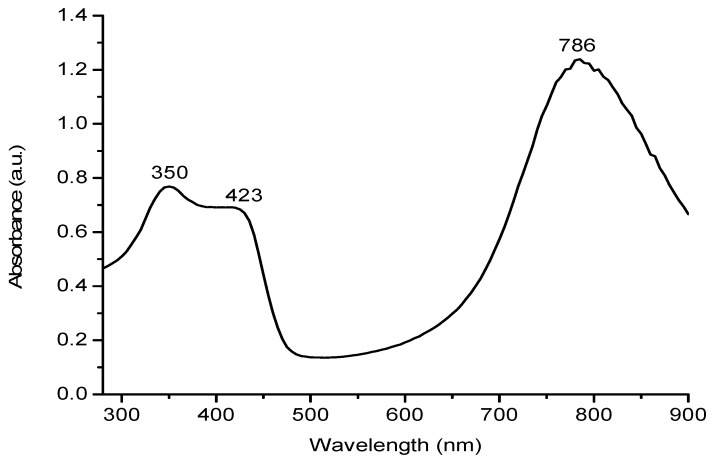
UV/Visible spectrum of PANI salt.

The UV/Visible spectrum of optimized PANI salt (Figure 5) exhibit all featured characteristics of good quality PANI. 

### 3.4. Extent of Doping

The extent of doping was calculated from the UV/Visible absorption spectra and is shown in Table 6. The level of doping is the ratio between exciton (π-polaron transition)/benzenoid (π–π* transition) [17]. A very slight difference has been observed in the extent of doping for different samples synthesized at different conditions. These results suggest that the extent of doping into PANI chain is not influenced by changing the amounts of monomer, dopant and solvent, however PANI B9 shows lower level of doping which might be a result of over oxidation of PANI due to more oxidant providing less chance to the dopant to enter into a polymer chain [31].

### 3.5. FTIR Spectroscopy

**Figure 6:**
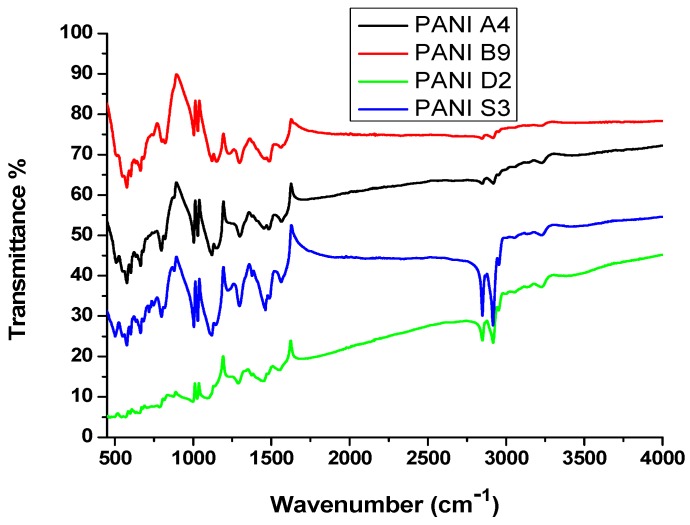
FTIR spectrum of different PANI samples as indicated.

**Figure 7:**
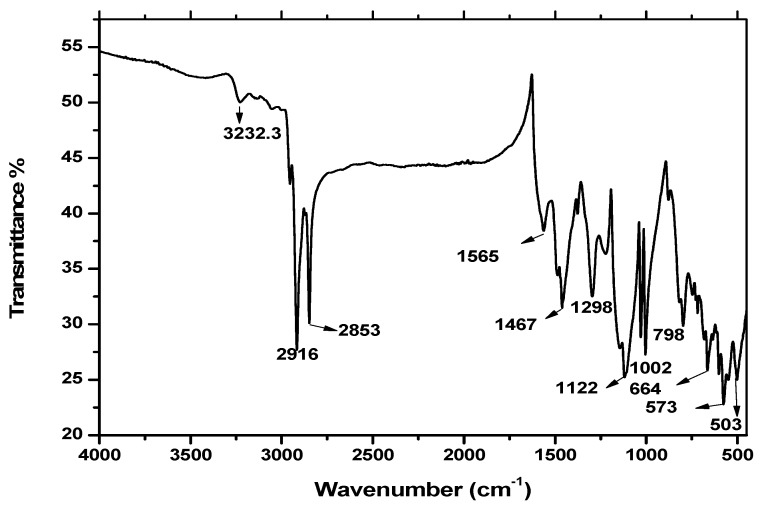
FTIR spectrum of PANI salt.

The FTIR spectra of different PANI samples, i.e., PANI A4, PANI B9, PANI D2 and PANI S3 are shown in Figure 6, where as the FTIR spectra of the PANI salt is shown in Figure 7. The peak positions of PANI along with their assignment are given in Table 7, where the 503 and 573 cm^−1^ band can be assigned to the absorption of –SO_3_H and SO_3_^−1^ [16,18].

The peak at 798 cm^−1^ represents the C-H bending vibrations [20], 1002 cm^−1^ is because of the absorption of –SO_3_H which is a prominent peak in all these samples [32]. The presence of bands at 503, 573 and 1002 cm^−1^ indicate the presence of DBSA. The peak at 1122 cm^−1^ is due to the in-plane bending vibration of C–H [33], while the 1298 cm^−1^ is because of C–N stretching of the benzenoid ring of PANI [34]. The band at 1467 cm^−1^ and 1565 cm^−1^ are assigned to benzenoid and quinoid ring vibration [14]. The presence of a peak at 3232 cm^−1^ confirmed the presence of N–H and NH_2_, which give rise to symmetric and asymmetric stretching [6]. The C–H stretching vibrations of the aromatic aniline ring are observed in the range of 2853 and 2916 cm^−1^ [8]. The appearance of all these typical peaks of PANI and DBSA provides the evidence for formation of PANI doped with DBSA.

### 3.6. X-Ray Diffraction Analysis

**Figure 8:**
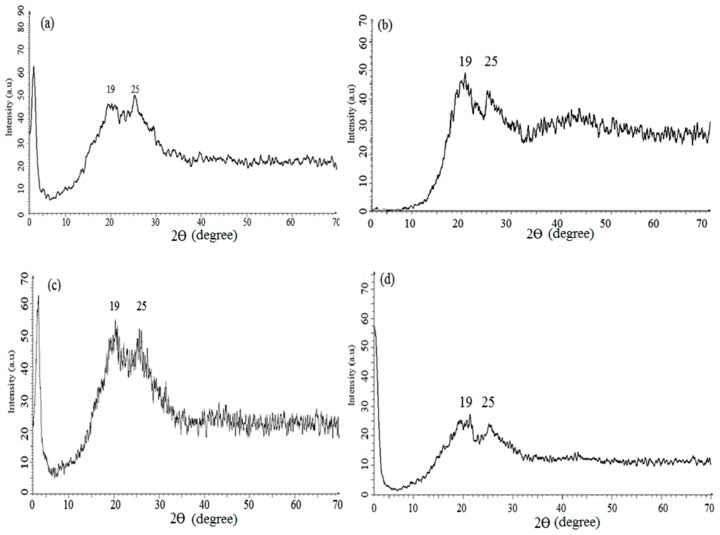
X-ray diffractograms of (**a**) PANI A4 (**b**) PANI B9 (**c**) PANI D2 (**d**) PANI S3.

**Figure 9:**
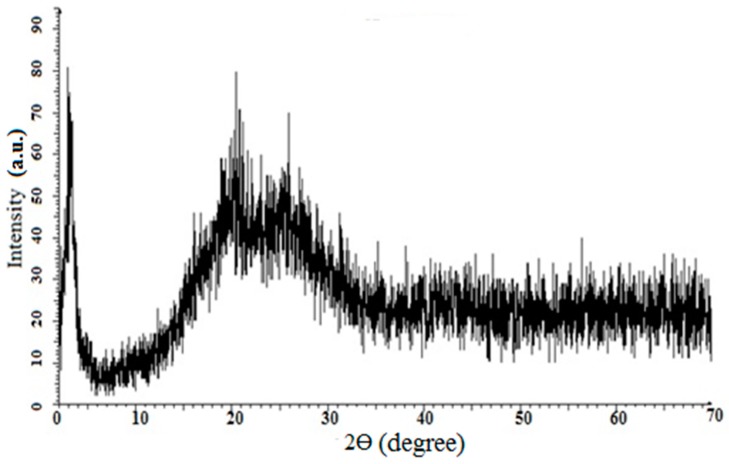
X-ray diffractogram of PANI salt.

The X-ray diffraction (XRD) technique is used to find the crystallinity of polymers in both the microcrystal and powder form. The XRD patterns of PANI A4, PANI B9, PANI D2, PANI S3 are given in Figure 8a–d, respectively, and that of PANI salt is presented in Figure 9. All these samples show a sharp peak at 25° and a broad peak at 19°, which are the characteristic peaks of PANI [35] The broad peak at 19° shows the amorphous nature of PANI which has strong correlation with the already reported one [15]. This peak clarifies the doping of polymer with DBSA. The intense peak at 25° indicates the presence of polymer crystallinity, which is due to the Vander Waal’s distance among the stacks of phenylene ring of PANI chain [9]. The crystallinity of PANI, due to repetition of quinoid and benzenoid rings in the polymer chain [36] leads to high conductivity of PANI salt.

### 3.7. Cyclic Voltammetry

**Figure 10:**
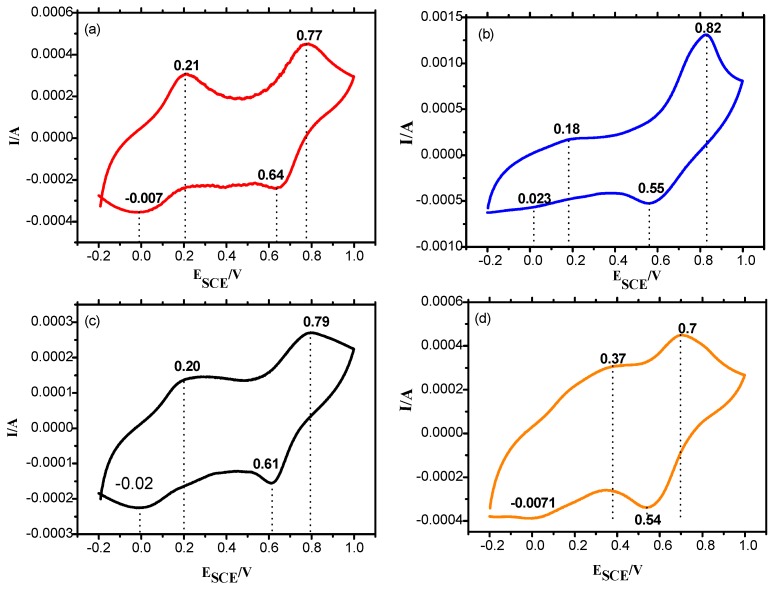
CV of synthesized PANI samples coated on a gold sheet electrode, in 0.5 M H_2_SO_4_ at a scan rate of 50 mV/s (**a**) PANI A4 (**b**) PANI B9 (**c**) PANI D2 (**d**) PANI S3.

**Figure 11:**
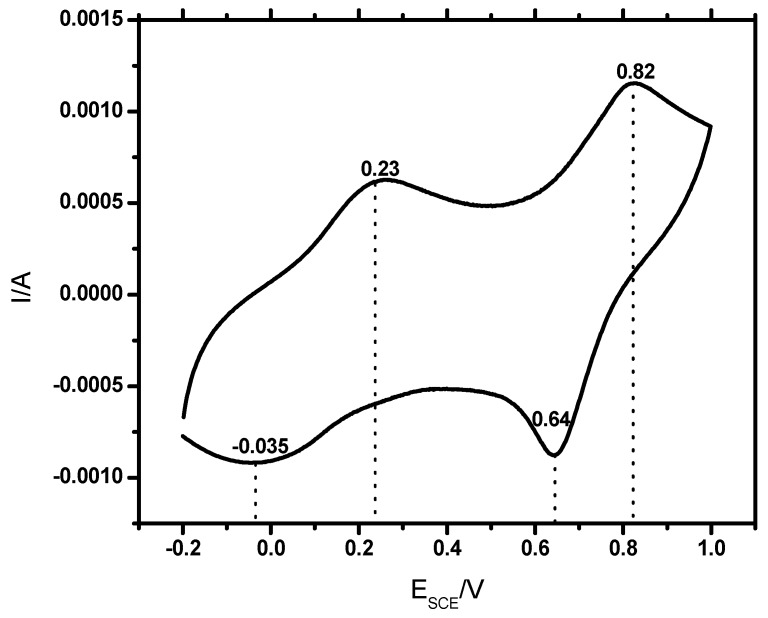
Cyclic voltammogram of PANI salt coated on a gold sheet electrode, in 0.5 M H_2_SO_4_ at a scan rate of 50 mV/s.

The redox nature of PANI salt was investigated through cyclic voltammetry, using gold as a working electrode in a potential range of −0.2 to 0.9 V, scan rate of 50 mV/s in supporting electrolyte of 0.5 M H_2_SO_4_. The cyclic voltammograms registered for PANI A4, PANI B9, PANI D2, PANI S3 and PANI salt are shown in Figure 10 and Figure 11. These cyclic voltammograms indicate that almost all samples have four peaks and two redox pairs. The first peak at around E_SCE_ = 0.18 to 0.23 V is due to the conversion of neutral leucoemeraldine form to partially oxidized emeraldine form of PANI. This peak is shifted to 0.37 V in the PANI S3 sample. The peak at around E_SCE_ = 0.77 to 0.82 V for all these samples can be assigned to the conversion of emeraldine to pernigraniline state of PANI. This peak has been shifted to 0.70 V in the PANI S3 sample. The shifting of these redox peaks may be due to the high doping level of DBSA content. Doping has a direct relation with conductance of polymer to some extent but beyond 50% doping, the excess of a long chain dopant such as DBSA causes steric hindrance. Consequently the interaction of PANI chain with DBSA decreases, which reduces the delocalization of π electrons and hence results in a low charge transfer [17]. In the reverse process, the peak at around E_SCE_ = 0.64 to 0.54V show the conversion of pernigraniline to emeraldine form of PANI. Similarly, the appearance of peaks at E_SCE_ = −0.007 V, 0.023 V, 0.0011 V, −0.0071V, and −0.035 V show the conversion of emeraldine form of PANI back to the fully reduced leucoemeraldine [35]. All these discrete peaks represent good electroactivity and reversibility of the material.

### 3.8. Application of the Synthesized PANI in Corrosion Protection of Steel

Literature studies reveal that electrochemically synthesized PANI has better corrosion protection ability when coated on metal surface in acidic media but fails to do so in saline media [32,37]. Rout, T.K. et al. used a formulated blend with polyaniline as a corrosion protecting material of steel and that reduced the corrosion of steel up to 3.5 mm/year, however due to lower solubility they used different materials for making blend which makes this material very expensive from an economic point of view [38].

Chemically synthesized PANI has also been used as a corrosion protective material for stainless steel [9].

**Figure 12:**
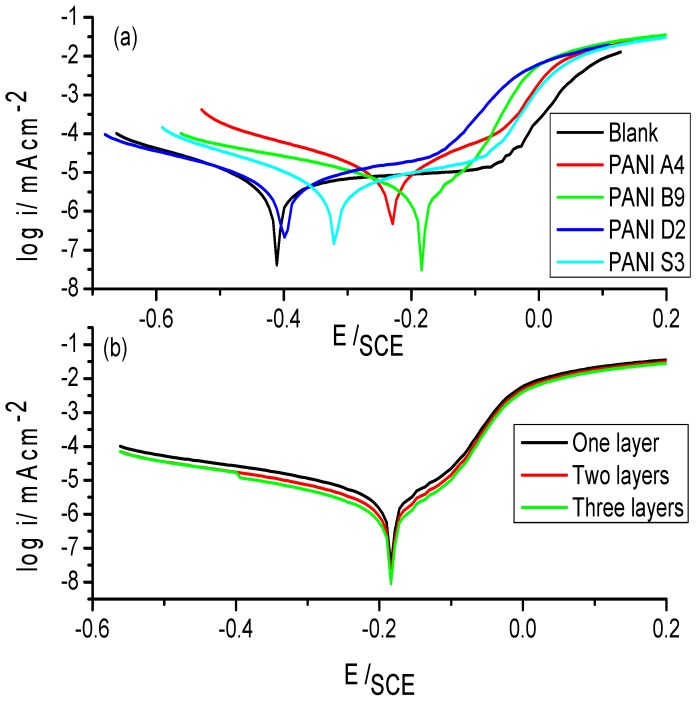
(**a**) Tafel plot of uncoated and different PANI coated stainless steel with different samples, as indicated. (**b**) Tafel plot of PANI coated via different no of drops on steel.

Keeping in mind the usefulness of PANI for corrosion protection of steel, the samples synthesized in present study were also tested for their corrosion protection ability. The Tafel plots of uncoated stainless steel and PANI A4, PANI B9, PANI D2 and PANI S3 coated stainless steel are shown in the Figure 12a. The corresponding values of *i*_corr_, E_corr_ and corrosion rate per year is shown in the Table 8. The Tafel plot of each optimized sample vs uncoated stainless steel shows corrosion protection on stainless steel in water samples from the Indian Ocean. The Indian Ocean has an average of 3.5% salinity, mostly due to NaCl. Generally the ocean environment is considered as a complex chemical system where the environment is more aggressive and corrosive than the laboratory prepared electrolytes. It possesses salinity, complex biological activity, a number of minor ions, pollutants and so on. However, it is the salinity which is considered as the major cause of corrosion in oceans. Yet it is not uniform even in the same ocean and there might be different ions content at different places. Generally, these ions of the dissolved salts, which mainly consist of NaCl, play a very important role in corrosion by covering the steel surface with insoluble products and enhancing passivation of the surface [39,40].

From the Table 8 it can be observed that among all samples of PANI the PANI B9 sample shows a remarkable positive potential shift and reduction in *i*_corr_ value as compared to uncoated stainless steel. The corrosion rate of uncoated stainless steel is 4.906 CR mm/year and that of PANI B9 is 1.575 CR mm/year showing that PANI coating on stainless steel has greatly reduced the corrosion of steel.

**Figure 13:**
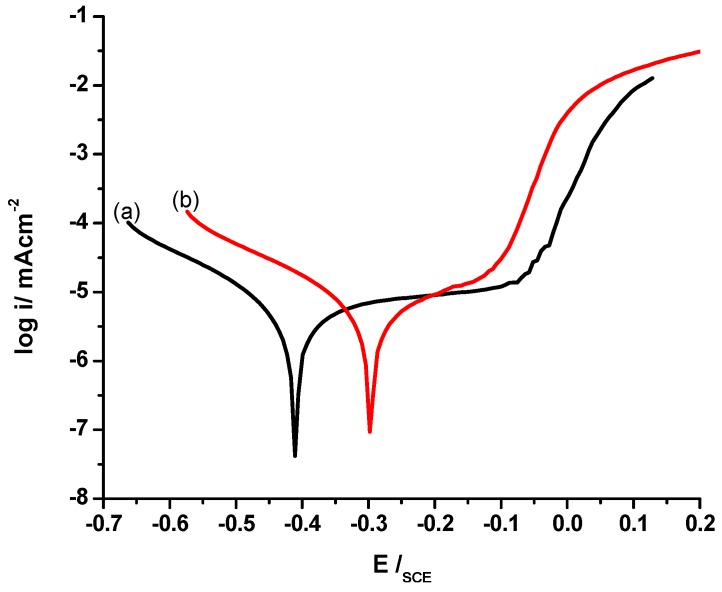
Tafel plot of (a) uncoated and, (b) PANI coated stainless steel.

Figure 13 shows the Tafel plot of PANI salt coated stainless steel vs uncoated stainless steel, and the corresponding *i*_corr_, E_corr_ and CR mm/year is shown in the Table 8. From the Table 8 it is clear that the values of *i*_corr_, E_corr_ and CR for uncoated stainless steel are 10.70 µA, −467.0 mV and 4.906 mm/year, respectively while for PANI salt coated stainless steel these values are 0.859 µA, −295.0 mV and 0.3927 mm/year, which indicate a remarkable positive shift in potential, great reduction in *i*_corr_ value and greater decrease in corrosion rate. These results indicate that diesel can effectively be used in the synthesis of efficient and cost effective corrosion protection coating.

Tafel polarization of single layered coating (Single drop coating) have been checked and compared with Tafel plots of many layered coatings (double and triple), as shown in the Figure 12b. A negligible difference in corrosion has been observed among single layered, doubled and tripled layered. However, it was found that sticking power of polymer was reduced in many layered coating as compared to a single layer.

### 3.9. Morphology of PANI

**Figure 14:**
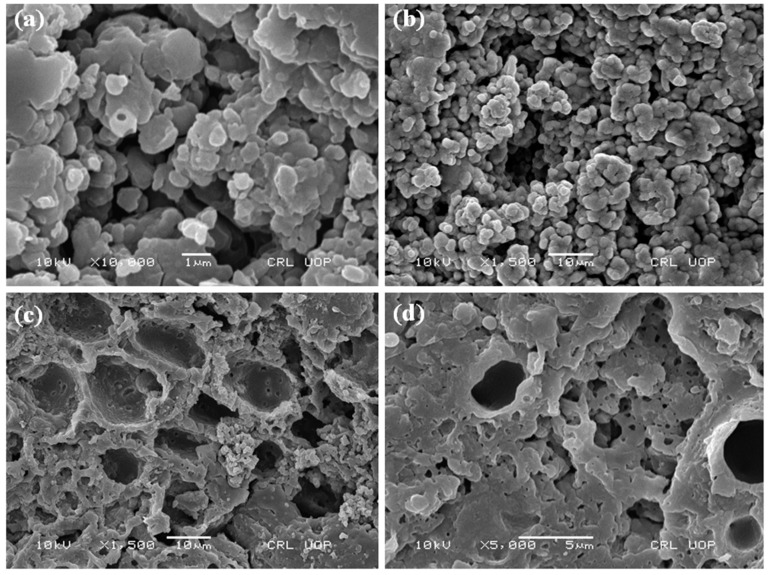
Scanning electron micrograph (**a**) PANI A4 (**b**) PANI B9 (**c**) PANI D2 (**d**) and PANI S3.

**Figure 15:**
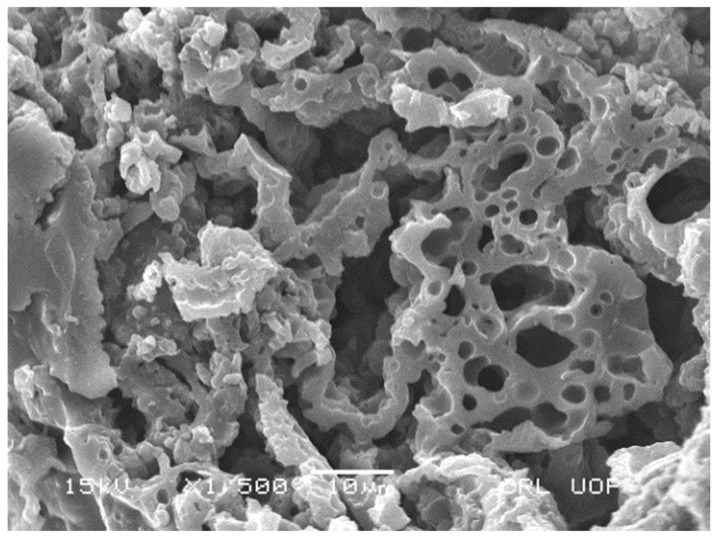
Scanning electron micrograph of PANI salt.

The morphology of PANI salt was studied at different magnification and is presented in Figure 14 and Figure 15. The images show that PANI salt has a cauliflower like morphology, having good porosity [17]. The appearance of porosity in all micrographs make the PANI salt a good corrosion inhibiting material having self healing ability due to presence of pores [41]. The material can also be used effectively for the immobilization of bio components.

### 3.10. Thermogravimeteric Analysis

**Figure 16:**
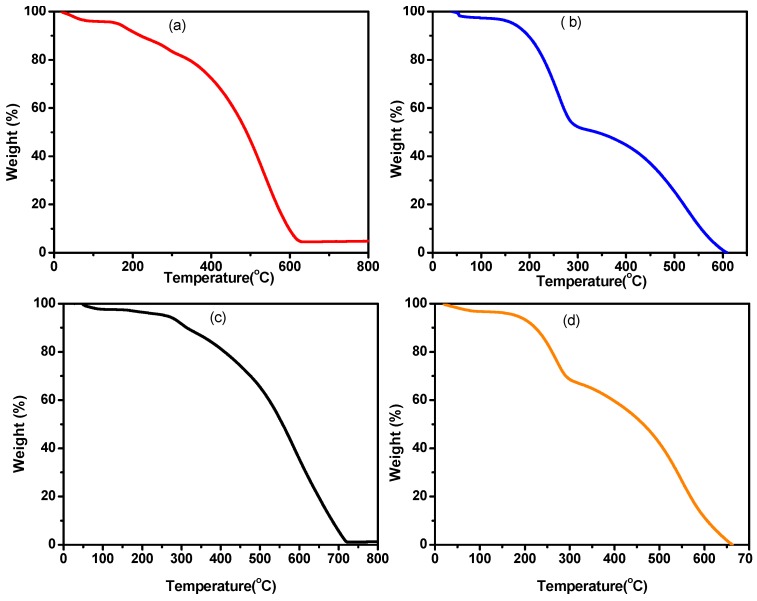
Thermograms of different (**a**) PANI A4 (**b**) PANI B9 (**c**) PANI D2 and (**d**) PANI S3.

**Figure 17:**
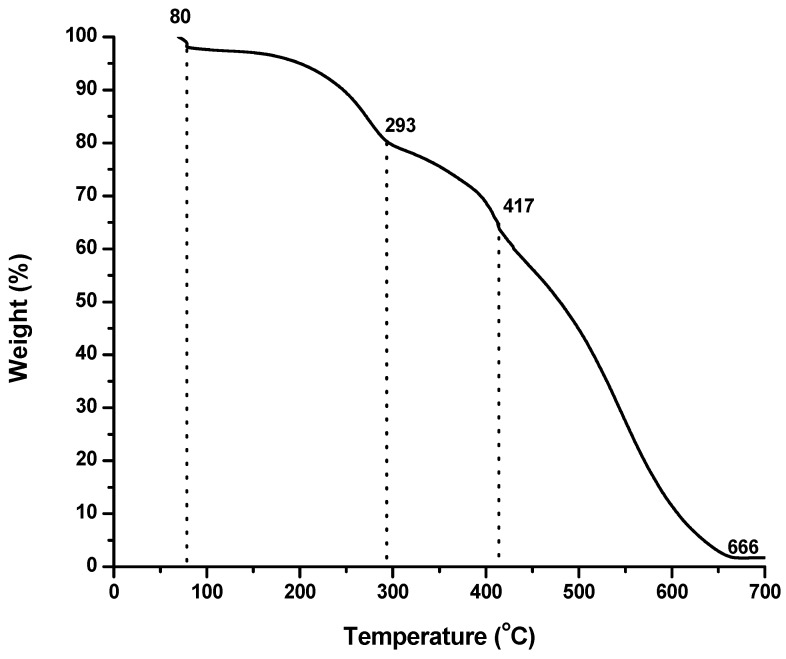
TGA curve of PANI salt.

Kinetics of thermal decomposition along with the quantity of dopant inserted in the polymer structural backbone is determined by thermogravimetric analysis (TGA). The thermograms of different samples of PANI are shown in Figure 16 and Figure 17. The sample was kept at 50 °C for one minute and then subjected to heat at the rate of 10 °C/min. The TGA curves shows that PANI A4 is thermally stable up to 305 °C, PANI D2 up to 293 °C, PANI B9 PANI up to 299 °C, PANI S3 PANI up to 300 °C and PANI salt up to 417 °C. The thermogram of PANI salt shows a three-step degradation pattern. The first weight loss occurs up to 293 °C which can be attributed to unbound moisture because PANI salt dried at vacuum still absorbs moisture due to its hygroscopic nature [2]. The second weight loss occurs at the range of 290–417 °C, which corresponds to the loss of dopant [28]. After 417 °C, the loss in weight occurs in a continuous way, which is due to the structural degradation of PANI backbone [42].

## 4. Conclusions

In conclusion, diesel can be effectively utilized for the cost effective synthesis of highly porous, soluble, thermally stable and anticorrosive polyaniline. The synthesized polymer retains all the good properties of PANI salt and hence make it a potential candidate for various technological applications. The cost of synthesis of PANI salt with improved solubility, thermal stability and good corrosion protection ability has been reduced to approximately 96% using diesel as a solvent instead of commonly used organic solvents.

## Figures and Tables

**Table 1:** Price of solvents in USD/L (2018/2019) and its effect on product properties.

Dispersion Medium	Price of Solvents in USD/L	Maximum % Yield	Thermal Stability (°C)	Solubility	Reference
Chloroform	13	52.4	480	NMP, DMSO, dimethylformamide (DMF)	[6]
Toluene	22	-	-	Toluene, Xylene	[11]
Toluene & iso octane	22 & 158	108–194	320–499	DMSO	[16]
Toluene & 2-propanol	22 & 25	61	-	Chlorofrom, 2:1 mixture of toluene & 2-propanol, NMP, Dichloromethane.	[7]
Chloroform & 2-butanol	13 & 22	25–30	500	2:1 mixture of toluene + 2-propanol, chloroform, DMSO & DMF.	[10]
*n*-hexane	12	81.97	320	-	[17]
Diesel	0.83	87.6	417	Chloroform, NMP, DMSO, 1:3 Mixture of toluene & 2-propanol	Present work

**Table 2:** Effect of amount of monomer (aniline) on percent yield of PANI.

SerialNo	mmol of Aniline	Weight of the PANI (g)	% yield	Sample Code
1	0.55	0.000	0.00	PANI A1
2	1.10	0.004	4.00	PANI A2
3	1.60	0.023	15.64	PANI A3
4	2.20	0.065	31.90	PANI A4
5	2.74	0.060	24.40	PANI A5
6	3.30	0.071	24.10	PANI A6
7	3.83	0.054	15.12	PANI A7

**Table 3:** Effect of amount of oxidant (Benzoyl peroxide) on percent yield PANI.

Serial No	mmol of Oxidant	Weight of PANI (g)	% yield	Sample Code
1	0.21	0.000	0.00	PANI B1
2	0.41	0.001	0.49	PANI B2
3	0.83	0.005	2.45	PANI B3
4	1.03	0.008	3.92	PANI B4
5	1.24	0.026	12.74	PANI B5
6	1.65	0.065	31.86	PANI B6
7	2.06	0.080	40.8	PANI B7
8	2.48	0.124	60.7	PANI B8
9	2.90	0.178	87.6	PANI B9
10	3.30	0.123	60.4	PANIB10

**Table 4:** Effect of amount of dopant (DBSA) on percent yield of PANI.

Serial No	mmoles of DBSA	Weight of PANI (g)	% yield	Sample Code
1	0.91	0.065	31.86	PANI D1
2	1.52	0.116	56.86	PANI D2
3	2.13	0.112	54.90	PANI D3
4	3.04	0.096	47.05	PANI D4
5	3.65	0.045	22.05	PANI D5
6	4.56	0.084	41.17	PANI D6

**Table 5:** Effect of amount of solvents (diesel/water) on percent yield of PANI.

Serial No	Amount of Organic Solvent (mL)	Amount of Water (mL)	Weight of PANI (g)	% Yield	Sample Code
1	50	10	0.026	12.574	PANI S1
2	40	20	0.042	20.58	PANI S2
3	30	30	0.043	21.07	PANI S3
4	20	40	0.030	14.70	PANI S4
5	10	50	0.036	17.64	PANI S5

**Table 6:** The extent of doping of DBSA into PANI Chain.

Serial No	Sample	E/B
01	PANI A4	2.21
02	PANI D2	2.25
03	PANI B9	2.14
04	PANI S3	2.22

**Table 7:** Peak assignments in the FTIR spectra of PANI salt.

Serial No	Peak Positions (cm^−1^)	Peak Assignment
01	503 and 573	–SO_3_H and SO_3_^−1^ of DBSA
02	798	Out of plane C–H bending vibration
03	1002	–SO_3_H of DBSA
04	1122	In-plane bending vibration of C–H
05	1298	C–N stretching of benzenoid ring
06	1467	Benzenoid ring
07	1565	Quinoid ring C–N strecting
08	2853 and 2916	C–H stretching vibrations of aromatic aniline ring
09	3232	Symmetric and asymmetric stretching of NH_2_ and NH

**Table 8:** Values of *i*_corr_ (µA), E_corr_ (mV), β_A_ (V/decade), β_C_ (V/decade) and CR (mm y^−1^) for different samples of PANI salt.

Material	*i*_corr_ (µA)	E_corr_ (mV)	β_A_ (V/decade)	β_C_ (V/decade)	CR (mm y^−1^)
Uncoated steel	10.70	−467.0	0.5304	0.2708	4.906
PANI A4	7.050	−231.0	0.1243	0.1818	3.223
PANI B9	3.450	−184.0	0.959	0.2586	1.575
PANI D2	4.870	−400.0	0.272	0.221	2.226
PANI S3	4.380	320.0	0.2865	0.1875	2.000
PANI salt	0.859	−295.0	0.1086	0.1194	0.3927
